# Medical management of genitourinary tuberculosis

**DOI:** 10.4103/0970-1591.42619

**Published:** 2008

**Authors:** Tamilarasu Kadhiravan, Surendra K. Sharma

**Affiliations:** Department of Medicine, All India Institute of Medical Sciences, New Delhi - 110 608, India

**Keywords:** Directly observed treatment, drug therapy, glucocorticoids, urogenital tuberculosis

## Abstract

Antimycobacterial chemotherapy is the mainstay of treatment for the majority of patients with genitourinary tuberculosis (GUTB). A large body of evidence from clinical trials suggests that short-course chemotherapy regimens, employing four drugs including rifampicin and pyrazinamide, achieve cure in most of the patients with tuberculosis (TB) and are associated with the lowest rates of relapse. Standard six-month regimens are adequate for the treatment of GUTB. Directly observed treatment, short-course (DOTS) is the internationally recommended comprehensive strategy to control TB, and directly observed treatment is just one of its five elements. DOTS cures not only the individual with TB but also reduces the incidence of TB as well as the prevalence of primary drug-resistance in the community. Corticosteroids have no proven role in the management of patients with GUTB. Errors in prescribing anti-TB drugs are common in clinical practice. Standardized treatment regimens at correct doses and assured completion of treatment have made DOTS the present-day standard of care for the management of all forms of TB including GUTB.

## INTRODUCTION

Tuberculosis (TB), a disease that is the cause of unaccountable human suffering and economic loss, paradoxically is to be considered a model disease as far as the scientific understanding of the disease *per se* is concerned. It is one of the earliest human afflictions for which a definitive cause was discovered. Tuberculosis is a disease for which the details regarding the epidemiology, transmission, pathogenesis, natural history, prevention, and treatment have been studied and understood in greater detail than any other human disease. In this era of evidence-based medicine, it is worthwhile to mention that the first-ever randomized controlled trial (RCT) in the history of modern medicine was conducted in the field of TB. In the judgment of Archie Cochrane, a father figure in the field of epidemiology, the specialty in medicine that deserves a gold medal, for being the most evidence-based, is TB.[1] Notwithstanding these distinctions, it is a sad fact that TB continues to be a major killer even today. In this review, we revisit the principles of the medical treatment of TB in general and also focus on the aspects of treatment specific to the management of genitourinary TB (GUTB).

## PRINCIPLES OF CHEMOTHERAPY OF TUBERCULOSIS

Short-course combination chemotherapy (SCC) is the standard of care for the treatment of TB.[2] Short-course combination chemotherapy is the logical culmination of a series of well-conducted clinical trials guided by simple yet elegant studies performed in vitro and in animal models on the effect of drugs on the growth of Mycobacterium tuberculosis. Before the advent of streptomycin, the treatment of patients with TB largely revolved around the concepts of “rest for the affected patient in sanatorium and rest for the affected portion of the lung by collapse therapy”.[3] Streptomycin was the first antibiotic discovered to have antimycobacterial activity. In 1948, streptomycin was demonstrated to be superior to bedrest alone for the treatment of patients with advanced pulmonary TB in an RCT conducted by the British Medical Research Council.[4] From a historical perspective, this trial is important for what streptomycin was unable to achieve rather than what streptomycin did achieve. Despite the impressive initial clinical improvement brought about by streptomycin, it was unable to achieve cure in these patients, and in fact 75% of patients developed drug-resistant strains within three months of treatment with streptomycin.[3,4] Thanks to this lesson, the problem of acquired drug-resistant, as we call today, thus came to be recognized very early in our efforts to treat TB.

What is the cause for this acquired drug-resistance? Resistance to an antibiotic is the result of mutations that occur spontaneously in a bacterial population. Mutation is a chance event occurring as a result of random error in DNA replication and does not really require prior exposure of the organism to the antibiotic concerned. What actually happens in acquired drug-resistance is, the wild drug-susceptible members of the bacterial population get killed by the antibiotic whereas a mutant member resistant to the antibiotic, if happens to be present in that population, grows unopposed and dominates the population. Thus, the antibiotic *per se* does not stimulate mutagenesis; rather, it provides a survival advantage to a naturally existing mutant organism if present. This is known as ‘antibiotic selection pressure.’ Since the development of a genetic mutation is a chance event, the factor that determines whether or not a mutant organism will be present in a population is the size of the population, bigger the population greater the chance that one of its members harbors a mutation.

Spontaneous mutations conferring resistance to isoniazid (INH) occur at a rate of 2.56 × 10^−8^ every generation of bacillary multiplication.[5] The rate of mutations to streptomycin and ethambutol are largely similar to that for INH-resistance. Rifampicin-resistance mutations occur at a substantially lower rate of 2.25 × 10^−10^ every generation.[5] Since the simultaneous occurrence of these mutations in a single bacillus is independent of each other, the rate of mutants resistant to two or more drugs can be obtained by multiplying the rates of individual mutations. Thus, the rate of combined resistance to INH and rifampicin would be 5.76 × 10^−18^ (2.56 × 10^−8^ multiplied by 2.25 × 10^−10^).

In a population of *M. tuberculosis* with multiple generations of bacilli, the ratio of drug-resistant to susceptible bacilli would be 1:10^6^ for INH, 1:10^8^ for rifampicin, and 1:10^14^ for combined resistance to both these drugs.[6] The bacillary load in an untreated patient with advanced pulmonary TB (cavitary disease) is in the order of 10^7^ to 10^9^ bacilli.[6] Thus, prior to treatment, a few of these bacilli would be resistant to one of the drugs while none of them would be resistant to two drugs simultaneously. When these patients are treated with a single drug only, under the selection pressure, these drug-resistant bacilli would emerge eventually resulting in treatment-failure. But, concomitant administration of a second drug would prevent the emergence of these drug-resistant bacilli and thereby averts treatment failure.

## EVOLUTION OF SCC

Para-aminosalicylic acid (PAS) was the second drug discovered to be active against *M. tuberculosis*. Subsequently, in 1952 it was demonstrated that the combination of PAS with streptomycin decreased the risk for streptomycin-resistance substantially.[7] In the same year, INH was introduced and was more effective than streptomycin and PAS. Over the next few years, several RCTs testing the triple drug combination of INH, streptomycin, and PAS were carried out.[8,9] Soon this regimen became the *de facto* standard for the treatment of TB. At about the same time, in 1959, a seminal study conducted in Southern India by the then Tuberculosis Chemotherapy Centre, Madras (currently Tuberculosis Research Centre, Chennai) demonstrated that treating patients with pulmonary TB at their homes was equally effective and did not entail an increased risk for TB among the household contacts.[10]

With these developments, new problems cropped up. The combination of INH, streptomycin, and PAS needed at least 12 months of treatment. First, it was costly, prohibiting its use in resource-poor countries.[3] Second, with the focus of treatment shifting from in-hospital observed treatment to domiciliary self-administered treatment, it was soon realized that non-adherence to prescribed treatment was a major obstacle to successful treatment.[11] As narrated by Mitchison, this problem was tackled using two approaches: i) development of intermittent regimens that could be much easily supervised by a healthcare provider, and ii) shortening the duration of treatment by incorporation of more potent newer drugs with good sterilizing activity.[3] Armed with laboratory evidence for good sterilizing activity of rifampicin and pyrazinamide, combinations containing these drugs were soon tested in RCTs conducted in East Africa and elsewhere, and it was conclusively demonstrated that six-month regimens containing rifampicin and pyrazinamide are very effective with fastest rates of culture-conversion and lowest rates of relapse.[12,13] These studies form the evidence base for the drug combinations used currently for the treatment of TB.

## RATIONALE OF MODERN DAY FOUR-DRUG REGIMEN

**Figure 1:**
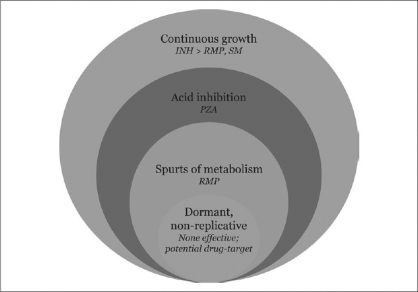
Bacillary Subpopulations in Active Tuberculosis - Mitchison's Hypothesis. Circles represent the subpopulations of *M. tuberculosis* that vary in metabolic activity and multiplication; size of the circles is not proportional to the actual size of the subpopulation. Drugs active against the respective subpopulation appear in italics. *INH* = isoniazid; *PZA* = pyrazinamide; *RMP* = rifampicin; *SM* = streptomycin[14]

The two most important facets of efficacy of any combination regimen for the treatment of TB are, i) rapid and complete killing of the bacillary population resulting in cure, and ii) prevention of relapse following successful cure. With a wealth of information obtained from clinical trials on the efficacy of various combinations regarding these outcomes it became evident, as far as the treatment of TB is concerned, all drugs are not made equal. It was Denis Mitchison who collated and intelligently interpreted these data. He hypothesized that the bacillary population is heterogeneous with respect to metabolic activity and multiplication.[14] Further, he inferred that there are at least four distinct subpopulations of bacilli in a patient with active TB, pulmonary TB being the prototype [Figure 1].

A large majority of bacilli are continuously multiplying and metabolically active corresponding to the log-phase growth characteristics *in vitro*. Isoniazid is the most active drug against these actively multiplying bacilli. Rifampicin and streptomycin are also effective in killing these bacilli. Ethambutol has a bacteriostatic effect on this subpopulation of bacilli. Another major subpopulation is constituted by the bacilli in acidic environments such as the caseous necrotic material of the cavities and inside the phagolysosomes of macrophages. Pyrazinamide is most active in such acidic milieu since it gets converted to its active form pyrazinoic acid in an acidic environment, where other drugs fail to act. The third subpopulation of bacilli remain metabolically inactive, but exhibit intermittent spurts of metabolism. Rifampicin due to its rapidity of action is very effective against these bacilli, killing them within the narrow window of opportunity during spurts of metabolic activity.

The fourth subpopulation, the most elusive, survives in a state of near-complete silence in terms of metabolic activity known as non-replicative persistence or dormancy, similar to that found in persons with latent TB infection. None of the currently available drugs are active against this small pool of bacilli. Their metabolic and growth characteristics are being unraveled by ongoing research at the molecular level, potentially opening up new avenues for therapeutic exploitation.[15]

Stated in a simplified way, INH kills rapidly the actively multiplying bacilli contributing much of the early bactericidal activity, while rifampicin and pyrazinamide kill the hidden strategic subpopulations of bacilli thereby sterilizing the lesions rapidly. The sterilizing activity of these two drugs is reflected as accelerated culture-conversion and markedly reduced risk of relapse following cure. The sterilizing activity of pyrazinamide is limited to the first two months of treatment, and it has been found that there is no appreciable benefit when pyrazinamide is continued beyond the first two months.[9]

As mentioned earlier, pyrazinamide is active against only a selected subpopulation of bacilli in the acidic milieu, and it is inherently ineffective against the rest of the bacillary population. In the presence of INH-resistance, rifampicin would be the only drug active against this latter population of bacilli, and this would amount to rifampicin monotherapy predisposing to development of rifampicin-resistance.^16^ In such a situation, a fourth drug such as ethambutol would act as a safeguard against the emergence of rifampicin-resistance. For this reason, it is recommended that in settings where the prevalence of primary resistance to INH is ≥4%, as is the case in India and other countries where TB is endemic, a fourth drug should be added to the intensive phase of the regimen.[16] After an intensive phase for two months, the size of the bacillary population gets reduced to a very small size. A combination of two drugs, INH and rifampicin, would suffice during the continuation phase, as the chances of developing resistance are very low. However, if either rifampicin or pyrazinamide is not included in the regimen, the continuation phase has to be prolonged beyond the usual four months duration.[17] The simple reason being there cannot be a six-month regimen that does not include both rifampicin and pyrazinamide.

## Directly observed treatment, short-course (DOTS) - The sure cure for tuberculosis

During the later part of the second half of the 20^th^ century the interest of health agencies, governments, and researchers started waning.[18] Some even believed that TB had been conquered, while it continued unnoticed to claim millions of lives in the developing and underdeveloped countries. Arrival of the human immunodeficiency virus (HIV) epidemic in the 1980s drew the attention of the global community to TB again. Finally, the apathy ended, and the World Health Organization (WHO) resolved in 1991 to detect 70% of infectious cases of TB and achieve cure in 85% of them adopting a new strategy subsequently labeled DOTS.[18] The WHO went on to declare TB a global emergency in the year 1993. DOTS (directly observed treatment, short-course) comprises five essential elements and directly observed treatment is just one among them [Table 1]. Consolidating the achievements of the DOTS strategy,[19] the WHO has expanded subsequently the scope and reach of TB control activities as envisaged in the Stop TB Strategy [Table 1].[20] In India, DOTS implementation began in the year 1993 on a pilot basis, and large-scale expansion of DOTS began in 1997 under the aegis of the Revised National TB Control Programme (RNTCP). By March 2006, 100% coverage of the nation had been achieved.[21] Till date, more than 6.7 million patients with TB have been started on DOTS, the treatment success rate has remained consistently above the global benchmark of 85%, and about 1.2 million lives have been saved.[21]

**Table 1:** Elements of DOTS and components of the Stop TB strategy

**DOTS**
Political commitment with increased and sustained financing
Case detection through quality-assured bacteriology
Standardized treatment with supervision and patient support
An effective drug supply and management system
Monitoring and evaluation system, and impact measurement
**Stop TB Strategy**
*Pursuing high-quality DOTS expansion and enhancement*
*Addressing TB/HIV, MDR-TB and other challenges*
- Implement collaborative TB/HIV activities
- Prevent and control MDR-TB
- Address prisoners, refugees, other high-risk groups and special situations
*Contributing to health system strengthening*
- Actively participate in efforts to improve system-wide policy, human resources, financing, management, service delivery, and information systems
- Share innovations that strengthen health systems, including the Practical Approach to Lung Health (PAL)
- Adapt innovations from other fields
*Engaging all care providers*
- Public-Public and Public-Private Mix (PPM) approaches
- Implement International Standards for TB Care
*Empowering people with TB, and communities*
- Advocacy, communication and social mobilization
- Community participation in TB care
- Patients’ charter for TB care
*Enabling and promoting research*
- Program-based operational research
- Research to develop new diagnostics drugs, and vaccines

Adapted from Reference[20]; TB = tuberculosis; MDR-TB = multidrug-resistant TB

The DOTS strategy is based on hard scientific evidence, and any apprehension regarding its efficacy is unfounded. Direct observation of treatment is the only way to ensure compliance with treatment. It is impossible to predict which patient will be adherent and who will be non-adherent. None of the factors such as age, gender, education, employment, profession, socioeconomic status, and marital status help the physician reliably predict non-compliance. Supervised administration of drugs is easier with intermittent treatment, and intermittent regimens are more cost-effective than daily regimens especially in resource-limited settings.

Intermittent regimens have been compared head-to-head with daily treatment in several RCTs and have been shown to be as effective as the latter.[22,23] Evidence from animal models suggests that intermittent treatment is in fact more efficacious that daily treatment.[14] The reason for this might lie in post-antibiotic effect (PAE). PAE is the continued suppression of bacterial multiplication even after the level of the drug has fallen below the therapeutic concentration. The PAE of INH lasts for about 18 h and that of rifampicin lasts for about 68 h. A combination of INH and rifampicin is synergistic and inhibits the multiplication of *M. tuberculosis* for about 160 h following a single exposure.[24] Though not well documented in the literature, it is generally accepted that drug-induced hepatotoxicity occurs less commonly in patients treated with intermittent regimens. The relative risk of immune-mediated reactions to rifampicin is slightly higher with intermittent treatment.[25] However, the absolute risk of such an adverse effect is very low, and the benefits of DOTS clearly outweigh any such risk.

The greatest testimony to the effectiveness of the DOTS strategy comes from epidemiologic data from countries with sustained implementation of DOTS. In China, implementation of DOTS over a period of 10 years has been shown to have resulted in a 30% decrease in the incidence of TB cases as well as primary drug-resistance.[26] Similar observations have been made from Mexico. In addition, a decrease in the incidence of multidrug-resistant TB (MDR-TB) was also observed in Mexico.[27] Thus, the DOTS strategy not only has the maximum efficacy to cure an individual with TB, it has the potential to halt the progress of TB in the community as well and reverse the epidemiologic trends. For the first time ever in the year 2005, TB incidence rates have been found to have stabilized or declining in all the six WHO regions.[20] However, the total number of cases is still increasing attributable to population growth, and the task of controlling TB is far from complete; rather, it has just begun.

## ISSUES SPECIFIC TO GUTB

### How long to treat?

The principles of treatment of GUTB are no different from that for a case of smear-positive pulmonary TB. Even though other forms of extrapulmonary TB such as TB lymphadenitis and minimal to moderate unilateral pleural effusion are considered milder forms of the disease, GUTB is to be considered a severe form of TB for two reasons. First, in renal TB the estimated size of the bacillary population is similar to that of cavitary pulmonary TB (10^7^-10^9^).[16] Second, if not aggressively treated, GUTB could potentially result in irreversible structural and functional damage to the organs involved.

It is a common practice for clinicians to treat GUTB for periods longer than six months. Till date, no RCT has been conducted to address the issues involved in the management of GUTB. However, there is no reason why the standard six-month regimen may be inadequate to treat patients with GUTB. Recommendations by the American Thoracic Society/Centers for Disease Control/Infectious Diseases Society of North America, British Thoracic Society, WHO, International Union Against Tuberculosis and Lung Disease, and European Association of Urology all state that the standard six-month SCC is effective for the treatment of patients with GUTB.[16,17,28–30] Details of the regimens followed in the RNTCP in accordance with the WHO recommendations are given in Table 2.

**Table 2:** Treatment regimens used in the revised national tuberculosis control programme (RNTCP)

Treatment category/definition	Treatment regimen*
Category I†	2H_3_R_3_Z_3_E_3_§ + 4H_3_R_3_
New sputum-smear positive	
Seriously-ill sputum-smear negative	
Seriously-ill extrapulonary‡	
Category II	2H_3_R_3_Z_3_E_3_S_3_ + 1H_3_R_3_Z_3_E_3_§ + 5H_3_R_3_E_3_
Sputum-smear positive relapse	
Sputum-smear positive failure	
Sputum-smear positive treatment after default	
Others‖	
Category III	2H_3_R_3_Z_3_ + 4H_3_R_3_
New sputum-smear negative, not seriously ill	
New extrapulmonary, not seriously ill	

Adapted from Reference[21]; E = ethambutol, 1200 mg/dose; H = isoniazid, 600 mg/dose; R = rifampicin, 450 mg/dose (patients weighing 60 kg or more receive an additional dose of 150 mg); S = streptomycin, 750 mg/dose (500 mg/dose for those aged more than 50 years); Z = pyrazinamide, 1,500 mg/dose; dosage in patients weighing less than 30 kg and children is calculated according to body weight

* Numbers preceding the letters represent the duration of treatment in months; numbers in subscript represent the number of doses per week

† Includes all HIV co-infected patients irrespective of sputum-smear status, type of disease, and severity of HIV-related immunosuppression

‡ Includes all patients with meningeal, pericardical, genitourinary, spinal, or disseminated involvement, bilateral pleural effusions, or massive unilateral pleural effusion

§ If the patient remains sputum-smear positive at the end of intensive phase, then intensive phase has to be extended by one month

‖ Includes sputum-smear negative or extrapulmonary relapse or failure; should be supported by culture or histopathological evidence of disease activity

## What is the role of corticosteroids?

Another area of controversy in the treatment of GUTB is the utility of corticosteroids in the prevention of complications such as ureteric stricture/fibrosis. In the absence of any RCT on this issue, it is worthwhile to know what is known regarding the beneficial effects of corticosteroids in other forms of extrapulmonary TB. The only two clinical indications for which the use of corticosteroids has been demonstrated to improve the outcome are TB meningitis and pericardial TB.[17] Intriguingly, the benefit is evident in terms of mortality only. Use of corticosteroids does not reduce the development of complications such as constrictive pericarditis or neurological impairment.[31,32] Drawing parallels from these observations, it seems unlikely that corticosteroids would be able to reduce the development of complications such as ureteric obstruction in patients with GUTB. This issue is worth investigating.

## Dose modification in renal failure

A patient with obstructive uropathy due to GUTB may develop renal failure. On the other hand, patients with chronic renal failure and renal transplant recipients comprise a high-risk group for developing TB. In such situations, the dosage of anti-TB drugs would need to be modified according to the creatinine clearance. Doses of INH, rifampicin, and pyrazinamide need no adjustment in a patient with renal failure since these drugs are either eliminated almost entirely through biliary secretion or metabolized to non-toxic compounds.[28] The dosing interval for ethambutol has to be doubled in patients with advanced renal failure (creatinine clearance < 10 mL/min). Intermittent dosing regimens have not been adequately studied in the setting of renal failure. Patients with renal failure may develop peripheral neuropathy with INH more commonly and hence should receive pyridoxine supplementation (10 mg/day) as a preventive measure. Other common side-effects of first-line anti-TB drugs are listed in Table 3.[33]

**Table 3:** Important side-effects of first-line anti-tuberculosis drugs

Drug	Side-effects
Isoniazid	Hepatitis, peripheral neuropathy, systemic hypersensitivity with rash and fever, psychosis, convulsions, disulfiram-like reaction with alcohol
Rifampicin	Flu-like symptoms, nausea, anorexia, diarrhea, red-orange discoloration of secretions and contact lenses, hepatitis, cholestasis, thrombocytopenia, renal failure
Pyrazinamide	Nausea, anorexia, asymptomatic hyperuricemia, joint pains,*
Ethambutol	Retrobulbar optic neuritis, asymptomatic hyperuricemia, peripheral neuropathy
Streptomycin	Vestibular dysfunction, hearing loss, non-oliguric renal failure

Based on Reference[33]

* Polyarthralgias are common in patients receiving pyrazinamide; this is unrelated to hyperuricemia. Clinically manifest gout is rare

## EVALUATION OF A PATIENT WITH GUTB-A PHYSICIAN'S PERSPECTIVE

All patients with GUTB should be evaluated for concomitant involvement of the lungs as well as other organs. Review of symptoms such as cough, expectoration, hemoptysis, and dyspnea followed by a chest radiograph and examination of at least three sputum-smears for acid-fast bacilli is the minimum evaluation for pulmonary involvement required in all patients with GUTB. Patients should be meticulously questioned about treatment for TB in the past for a period more than one month. If present, the chances of drug-resistant TB are higher, and Category II DOTS would be the appropriate treatment [Table 2]. In India, about 5.2% of patients with TB have underlying HIV co-infection[20,34] and extrapulmonary involvement occurs more commonly among HIV co-infected patients.[35] All patients should therefore be offered voluntary counseling and testing services for detecting HIV co-infection. Data on GUTB in HIV co-infected patients are few. Future studies should focus on this subgroup of patients with GUTB.

The most common reason for planned treatment interruption is the development of drug-induced hepatotoxicity. Old age, malnutrition, hypoalbuminemia, and alcoholism are associated with increased risk of drug-induced hepatotoxicity.[36] All patients should have their liver function evaluated before initiation of treatment. Liver functions have to be periodically monitored in patients with preexisting liver disease, abnormal liver function at baseline, alcoholics, and those developing symptoms suggestive of drug-induced hepatotoxicity such as anorexia, vomiting, jaundice, unexplained fever, or epigastric pain.

## APPROACH TO A PATIENT WITH UNRESPONSIVE DISEASE

A patient with GUTB or any other form of TB who fails to show improvement with treatment, of symptoms or lesions evident on imaging, is a commonly encountered situation in clinical practice. It is pertinent in this regard to ask, ‘what is the definition of a delayed response?’ The answer to this question is very elusive: mycobacterial culture provides the most definitive answer. Unfortunately, mycobacterial culture is often not utilized in decision-making by the clinician. Even when performed, the time taken for the culture results to become available impedes decision-making in real time. Often, imaging is used as a surrogate to assess the response to treatment in these patients. Imaging studies have certain limitations in this regard. First, imaging findings are the last to improve with treatment and lag behind culture-conversion and clinical symptoms. Second, some of the findings on imaging such as fibrosis/scarring are irreversible changes and are not to be expected to regress completely with treatment. Clinicians should remember the fact that the aim is to treat the disease, not the films!

Judgment regarding delayed response to treatment remains largely subjective at least in the case of extrapulmonary TB. Nevertheless, certain rules of thumb can offer some help to the frustrated clinician. The following possibilities need to be considered - Is the regimen and dosage of drugs correct? Is the patient adherent? Was the diagnosis of TB correct? Is it drug-resistant TB? When all these possibilities have been reasonably excluded, the most likely explanation would be a delayed response to treatment which is a common phenomenon in extrapulmonary TB.

Another possibility to be considered is that of a paradoxical reaction or immune reconstitution inflammatory syndrome (IRIS). Paradoxical reaction or IRIS is the unexpected worsening of preexisting lesions and/or appearance of new lesions such as serosal effusions, pulmonary infiltrate, lymphadenopathy, intracranial lesions or fever in a patient with TB while receiving adequate treatment.[37] Again, a diagnosis of IRIS requires the exclusion of all the four possibilities stated above. Immune reconstitution inflammatory syndrome occurs more commonly among HIV co-infected patients with TB after the initiation of antiretroviral treatment. Immune reconstitution inflammatory syndrome is known to occur in HIV-negative patients with TB as well, albeit at a much lower frequency.

## COMMON ERRORS IN TREATMENT OF TUBERCULOSIS

Prescription errors are very common in clinical practice.[38,39] Prasad *et al.*, found that 75% of prescriptions for TB by private practitioners were erroneous; nearly half the patients were prescribed regimens which are not recommended by the WHO, and in 30% the doses were inappropriate.[38] Such errors are effectively eliminated by placing all patients with TB on DOTS which employs standardized regimens at correct doses. It needs to be emphasized that initiation of a fifth drug such as streptomycin or a fluoroquinolone along with the standard four-drug SCC, as practiced widely, confers no extra benefit to treatment-naive patients with any form of TB including GUTB. Another important error is the addition of a single drug to an apparently failing regimen.[39] Such a practice will only promote the emergence of resistance to the drug added, will not contribute meaningfully to achieve cure and hence is strongly discouraged.

Often, the newer fluoroquinolones such as levofloxacin, gatifloxacin, and moxifloxacin are used in the community setting for the empirical treatment of presumed urinary tract infections. These drugs have excellent antimycobacterial activity and hence may mask the presentation of GUTB thereby delaying the diagnosis.[40] Moreover, even short duration (less than two weeks) exposure of *M. tuberculosis* to fluoroquinolones could result in emergence of resistance. For these reasons, empirical use of fluoroquinolones should be avoided when GUTB is among the differential diagnoses.

## CONCLUSIONS

Chemotherapy of TB has become very efficacious over the years. DOTS is the most effective way of achieving cure in a patient with TB and is considered the standard of care. Standard Category I regimen is effective for the treatment of patients with GUTB. Currently, there is no evidence to recommend the use of corticosteroids in the management of patients with GUTB. The issue whether corticosteroids are effective in preventing the development of complications such as urinary obstruction merits investigation in future clinical trials.
